# Metagenomic sequencing reveals structural and functional differentiation of the rhizosphere bacterial communities associated with available potassium in *Atractylodes lancea* affected with root rot

**DOI:** 10.3389/fmicb.2026.1923567

**Published:** 2026-08-19

**Authors:** Li Li, Ruibo Liu

**Affiliations:** College of Modern Agriculture and Biological Engineering, Qinling Mountains Institute of Biological Resources, Shangluo University, Shangluo, Shaanxi, China

**Keywords:** community functional differentiation, high-throughput sequencing, microbiota imbalance, rhizosphere microbiota, soil physicochemical properties, traditional Chinese medicine

## Abstract

**Background:**

*Atractylodes lancea* is an economically valuable medicinal herb indigenous to China, and its yield and quality are severely threatened by root rot disease. The rhizosphere microenvironment plays a critical role in plant health. Yet, its relationship with root rot in *A. lancea* is poorly understood.

**Methods:**

This gap was addressed by collecting rhizosphere soils from healthy *A. lancea* plants and those infected with root rot. The physicochemical properties of the soil were determined, and metagenomic sequencing was performed to determine differences in the diversity, structure, composition, and functional characteristics of the rhizosphere bacterial communities between the two groups.

**Results:**

Compared with healthy plants, the contents of total nitrogen, total potassium, and available potassium in the rhizosphere soil of diseased plants increased significantly, by 8.11, 3.42, and 38.66%, respectively. Concurrently, the bacterial community diversity increased significantly, and the community structure exhibited an obvious separating trend between the two groups, with a marginally non-significant difference (*P* = 0.098). *Pseudomonadota, Streptomyces*, and *Trinickia* were relatively more abundant in the healthy group, while *Acidobacteriota, Cyanobacteriota, Gemmatimonadota, Gemmatimonas*, and *Sphingomicrobium* were significantly enriched in the diseased group according to independent samples Student's *t*-tests (*P* < 0.05). LEfSe analysis (LDA score > 4) revealed that all the differential genera in the healthy group belonged to the Burkholderiaceae family within *Pseudomonadota*. Functional prediction demonstrated that rhizosphere bacteria of healthy plants were predominantly enriched for genes involved in ABC transporter pathways, whereas diseased samples were enriched for secondary metabolite biosynthesis alongside significantly elevated abundance of auxiliary oxidoreductase genes. The abundance of auxiliary oxidoreductase genes was also significantly higher in the diseased group. Redundancy and correlation analyses showed that available potassium was strongly correlated with the divergence in the composition and function of the rhizosphere bacterial community.

**Conclusions:**

This study revealed that the occurrence of root rot was associated with imbalanced physicochemical properties of rhizosphere soil, shifts in bacterial community composition and structure, and alterations in metabolic functions of *A. lancea*. These findings elucidate rhizosphere responses linked to root rot and inform the sustainable cultivation of *A. lancea*.

## 1 Introduction

*Atractylodes lancea* (Thunb.) DC., a perennial medicinal herb in the family Asteraceae, is widely used in traditional Chinese medicine, in which its dried rhizome is utilized for drying dampness, invigorating the spleen, dispelling wind-cold, and brightening vision (Zhang et al., 2024). Modern pharmacological studies have demonstrated that *A. lancea* contains abundant bioactive compounds, including sesquiterpenes, enzymes, and polysaccharides, which exert multiple pharmacological activities, such as anti-inflammatory, anti-ulcer, hepatoprotective, and anti-tumor effects (Pi et al., 2024; Zhang et al., 2022).

The rhizosphere is a special microzone in the soil that is strongly affected by the activities of plant roots. It includes the root surfaces and adjacent soils. This zone represents the most active region for the exchange of materials between plants and soil, and it harbors one of the most complex microecosystems in nature (Orozco-Mosqueda et al., 2022). Changes in the physicochemical properties of the soil rhizosphere substantially influence the growth and health of plants and the diversity of soil microorganisms. Alterations in the soil pH, salinity, and content of nitrogen (N) can reshape the rhizosphere microbial community of American ginseng (*Panax quinquefolius*) and further disturb the growth and development of plants (Liu et al., 2021). Fierer and Jackson (2006) confirmed that the soil pH is closely associated with bacterial diversity, which peaks in neutral soils and declines in acidic ones. Rousk et al. (2009) provided additional detail, finding that fungi are more tolerant to acidic conditions than are bacteria, and as such, the ratio of the abundance of fungi-to-bacteria can increase nearly 30-fold when the soil pH decreases from 8.3 to 4.5. Accordingly, maintaining balanced soil physicochemical properties is essential for maintaining the health of plants and the diversity of the soil microorganisms.

Microbial diversity is a core factor that sustains the structural and functional stability of rhizosphere soil ecosystems (Compant et al., 2025). In addition, rhizosphere microorganisms can regulate the uptake of nutrients, disease resistance, and medicinal quality of healthful plants *via* their metabolic activities (Yu et al., 2022). Distinct differences in the microbial communities in the rhizosphere have been observed between healthy and diseased plants of various medicinal and agricultural crops. Deng et al. (2022) reported that rhizosphere microbial diversity of *Pinus koraiensis* increased markedly with aggravated pine wilt disease symptoms. A significant rhizosphere microecological imbalance was also found in Asian ginseng (*Panax ginseng*) suffering from rust root rot; there was a decrease in the fungal diversity and an increase in bacteria in the diseased soil (Wei et al., 2020). A previous study on *Schisandra chinensis* root rot revealed that the disease remarkably alters the diversity, structure, and functional profiles of the rhizosphere bacterial communities in this medicinal plant (Li et al., 2026). Collectively, soilborne pathogens can reshape the composition and structure of the rhizosphere microbiota and remodel the soil microenvironment. Nevertheless, systematic research linking the occurrence of root rot to rhizosphere microecological succession in *A. lancea* remains limited, and the underlying pathogenic mechanisms remain poorly understood.

Currently published research on *A. lancea* mainly focuses on pharmacological research on bioactive ingredients, including volatile oils and polysaccharides from its rhizome. These active components have a variety of biological impacts, such as anti-tumor and gastrointestinal regulatory effects (Lin et al., 2024; Zhou et al., 2024). In contrast, research on the cultivation of *A. lancea*, particularly systematic investigations on the microecology of its rhizosphere, remains limited. In recent years, frequent outbreaks of root rot have severely inhibited the growth of *A. lancea*, reduced its yield and quality for use as medicine, and become a major obstacle to its standardized cultivation. Herein, we collected rhizosphere soils from healthy *A. lancea* plants and those diseased with root rot and employed metagenomic sequencing to systematically compare the structure, composition, and functional traits of the rhizosphere microbial communities. This study aimed to reveal the intrinsic relationship between root rot and the microecological imbalance in the rhizosphere and provide an empirical basis and technical support for the green management of this disease and the high-quality cultivation of *A. lancea*. Specifically, this work fills a previously unreported knowledge gap by elaborating the mechanistic link connecting root rot occurrence, soil potassium dynamics, and functional shifts of rhizosphere bacterial communities in *A*. *lancea*.

## 2 Materials and methods

### 2.1 Site description

The field study was conducted from November 2021 to July 2025 at the *Atractylodes lancea* experimental base located in Guowan Village, Beikuangping Town, Shangzhou District, Shangluo City, Shaanxi Province, China (110°15′ E, 33°53′ N). It has an altitude of 1,104 m, an annual mean temperature of 12.8 °C, annual precipitation of 800 mm, and a frost-free period of approximately 202 days. As part of the Danjiang River watershed within the broader Yangtze River Basin, the area features high forest coverage and provides suitable mountainous conditions for the growth of *A. lancea*. Preparation of the land for this study was completed in autumn 2021. Before tilling, 9,000 kg·hm^−2^ of decomposed sheep manure and 380 kg·hm^−2^ of compound fertilizer were applied. Ridges 80-cm wide and 30-cm high were built. *Atractylodes lancea* seedlings were transplanted on the ridges in late November 2021 and spaced at 30 cm × 30 cm. The fields were managed according to standard local practices throughout the growing season, including regular manual weeding and shallow tillage. The soil moisture was maintained at an appropriate level, and drainage of the field was enhanced during the rainy seasons (July–September) to prevent waterlogging. No fungicides were applied during the entire growing season before the samples were collected.

### 2.2 Rhizosphere soil sampling

Sampling was conducted on July 25, 2025, which was the peak occurrence period of root rot and corresponded to the budding and early flowering stage of *Atractylodes lancea*. The five-point sampling method was used to establish sampling points. The five-point sampling method was used to establish sampling points. Healthy plants with vigorous growth, no visible disease symptoms on aboveground parts, and roots without blackening or rot were selected, while the selected diseased plants exhibited typical root rot symptoms, including leaf yellowing, defoliation, and underground root and rhizome rot. In total, 12 healthy plants with uniform growth and 12 diseased plants with similar growth status and consistent root rot severity were selected. Surface weeds and litter were removed before sampling. Sterile shovels were used to excavate the soil. The loose bulk soil around the root systems was removed, and the rhizosphere soil that tightly adhered to the root surfaces was then collected by shaking it from the roots. All the fresh samples were placed in insulated boxes with ice packs and transported to the laboratory within 10 h. The soil within 2 mm of the root surface was collected as the rhizosphere samples. After the stones, plant residue, and other debris had been removed, the samples were passed through a 2-mm nylon sieve and numbered.

Considering the high microenvironmental heterogeneity of field rhizosphere soil and individual differences among individual field-grown plants, the 12 rhizosphere soil samples from healthy plants were randomly pooled into three biological replicates (JZ-1, JZ-2, and JZ-3), such that four samples were combined to form one replicate. The 12 samples from the diseased plants were processed identically to obtain three biological replicates (BZ-1, BZ-2, and BZ-3). This pooling strategy was adopted to reduce random individual variation and background noise in metagenomic sequencing, thereby improving the representativeness of rhizosphere microbial characteristics at the field population level. Each composite sample was subsequently divided into two subsamples. One was stored at −80 °C for high-throughput sequencing, and the other was air-dried indoors at room temperature in order to analyze the physicochemical properties of the soil.

### 2.3 Determination of the soil physicochemical properties

The organic matter in the soil was determined by the potassium dichromate oxidation titration method (Hong et al., 2018). The total nitrogen (TN) was measured using the Kjeldahl method, and the hydrolyzable nitrogen (N) was analyzed *via* the alkaline hydrolysis diffusion method (Bremner, 1960). The available phosphorus (AP) and total phosphorus (TP) were quantified by the molybdenum-antimony colorimetric method (Qiao et al., 2018). The available potassium (AK) and total potassium (TK) were measured using flame photometry (Zebec et al., 2017).

### 2.4 DNA extraction and metagenomic sequencing

An E.Z.N.A.^®^ Soil DNA Kit (Omega Bio-tek, Norcross, GA, USA) was used to extract the total microbial genomic DNA from the soil according to the manufacturer's instructions. The concentration and purity of the extracted DNA were determined, and the DNA integrity was checked using 1% agarose gel electrophoresis. DNA was fragmented using a Covaris M220 system (Gene Company Limited, Hong Kong, China). DNA fragments of approximately 350 bp were recovered for paired-end (PE) library construction. Paired-end libraries were prepared using the NEXTFLEX Rapid DNA-Seq Kit (Bioo Scientific, Austin, TX, USA). Adapters containing complete sequencing primer annealing sequences were ligated to the blunt ends of the DNA fragments. Metagenomic sequencing was performed on an Illumina NovaSeq™ X Plus platform (Illumina, San Diego, CA, USA) by Shanghai Majorbio Bio-Pharm Technology Co., Ltd. (Shanghai, China). Adapter sequences at the 5′ and 3′ termini of raw reads were trimmed for quality control using fastp (Chen et al., 2018; v. 0.23.0; https://github.com/OpenGene/fastp). The resulting clean reads were subsequently *de novo* assembled with MEGAHIT (Li et al., 2015; v. 1.1.2; https://github.com/voutcn/megahit). Contigs with lengths of ≥300 bp were screened and retained as the final assembled sequences.

### 2.5 Bioinformatic analysis

#### 2.5.1 Gene abundance profiling

High-quality reads from each sample were aligned against the non-redundant gene set using SOAPaligner (v. soap2.21 release, https://github.com/ShujiaHuang/SOAPaligner) with a sequence identity threshold of 95%. The abundance of genes in each sample was calculated based on the alignment results (Li et al., 2008).

#### 2.5.2 Taxonomic and functional annotation

The amino acid sequences of the non-redundant gene set were searched against the NR, eggNOG, and Kyoto Encyclopedia of Genes and Genomes (KEGG) databases *via* BlastP using Diamond (v. 2.0.13, https://github.com/bbuchfink/diamond) with an e-value cutoff of 1E-5. The taxonomic source of each non-redundant gene was annotated based on the results of the NR database, and the taxonomic abundance was calculated by summing the abundance of the corresponding genes. The eggNOG database was used for Clusters of Orthologous Genes (COG) functional annotation, and the abundance of each COG group was obtained by accumulating the abundance of related genes. The KEGG database was used to functionally annotate the genes to determine their associated metabolic pathways (Buchfink et al., 2015).

The hmmscan tool (http://hmmer.janelia.org/search/hmmscan) was used to align amino acid sequences against the Carbohydrate-Active Enzymes (CAZy) database at an e-value of 1E-5 to obtain annotations of these enzymes. The total abundance of genes assigned to each enzyme family was defined as the abundance of the corresponding carbohydrate-active enzyme.

#### 2.5.3 Community diversity and differential analysis

Prior to all statistical analyses, the Shapiro–Wilk test and Levene's test were separately performed to test violations of the normality and homogeneity of variance assumptions of the subsequent parametric statistical analysis. The relative abundance of bacterial taxa at both the phylum and genus levels as well as KEGG pathways at Pathway Level 3 were quantified using the reads per kilobase of transcript per million mapped reads (RPKM) values. Differential abundance analysis of bacterial taxa was performed using Student's *t*-test with two-tailed testing. The false discovery rate (FDR) was employed for multiple hypothesis testing correction to eliminate false positive results associated with a large number of simultaneous comparisons, and the significance threshold was set at FDR < 0.05. The alpha-diversity indices, redundancy analysis (RDA), and visualization were performed using mothur (v. 1.30.2, https://mothur.org/wiki/calculators/) combined with R 3.3.1. The beta-diversity distance matrix was generated using QIIME 2020.2.0. RDA was performed to explore the relationships between phylum-level bacterial community composition and soil environmental factors, and 999 permutations were evaluated to test the statistical significance of environmental constraints. A Linear discriminant analysis Effect Size (LEfSe) analysis (http://galaxy.biobakery.org/) was performed to screen differential biomarkers based on community composition and functional profiles between groups. Briefly, the Wilcoxon rank-sum test was first adopted to identify taxa or KEGG pathways with significantly different abundance across groups, and linear discriminant analysis (LDA) was subsequently applied to quantify the effect size of each differential feature. Bacterial taxa satisfying Wilcoxon test *P* < 0.05 and logarithmic LDA score > 4.0 thresholds were defined as group-specific differential biomarkers, whereas KEGG functional pathways meeting threshold criteria of Wilcoxon test *P* < 0.05 and logarithmic LDA score > 2.5 were determined to be significantly differential pathways.

### 2.6 Statistical analysis

All the physicochemical data are presented as the mean ± SD. Differences between the two groups were assessed using an independent samples *t*-test. All statistical analyses were performed with SPSS 26.0 software (IBM, Armonk, NY, USA). A value of *P* < 0.05 was considered statistically significant.

## 3 Results

### 3.1 Soil physicochemical properties of the rhizosphere

Compared with the healthy group, the contents of TN, TK, and AK increased significantly in the rhizosphere soil of diseased *A. lancea* plants (*P* < 0.05; Table 1). Specifically, TN increased from 0.74 ± 0.02 g/kg to 0.80 ± 0.02 g/kg, by approximately 8.11%; TK increased from 18.40 ± 0.10 g/kg to 19.03 ± 0.31 g/kg, by about 3.42%; and AK rose sharply from 59.50 ± 2.55 mg/kg to 82.50 ± 2.35 mg/kg, by roughly 38.66%. No significant differences were detected in organic matter, total phosphorus, hydrolyzable nitrogen, or available phosphorus between the two treatments (*P* > 0.05). Such obvious enrichment of rhizosphere nitrogen and AK suggests that root rot disease is accompanied by abnormal accumulation of key mineral nutrients in the rhizosphere microenvironment.

**Table 1:** Physicochemical properties of rhizosphere soil from healthy and diseased *Atractylodes lancea*.

Treatment group	Organic matter (g/kg)	Total nitrogen (g/kg)	Total phosphorus (g/kg)	Total potassium (g/kg)	Hydrolyzable nitrogen (mg/kg)	Available phosphorus (mg/kg)	Available potassium (mg/kg)
JZ	11.43 ± 0.15a	0.74 ± 0.02b	2.56 ± 0.31a	18.40 ± 0.10b	57.53 ± 1.43a	30.27 ± 1.16a	59.50 ± 2.55b
BZ	11.70 ± 0.36a	0.80 ± 0.02a	2.32 ± 0.07a	19.03 ± 0.31a	58.00 ± 1.91a	30.07 ± 1.96a	82.50 ± 2.35a

Data are expressed as mean ± standard deviation values; different lowercase letters within the same column indicate significant differences at P < 0.05. JZ, healthy group; BZ, diseased group.

### 3.2 Diversity of the rhizosphere bacterial communities

#### 3.2.1 Alpha-diversity

**Figure 1:**
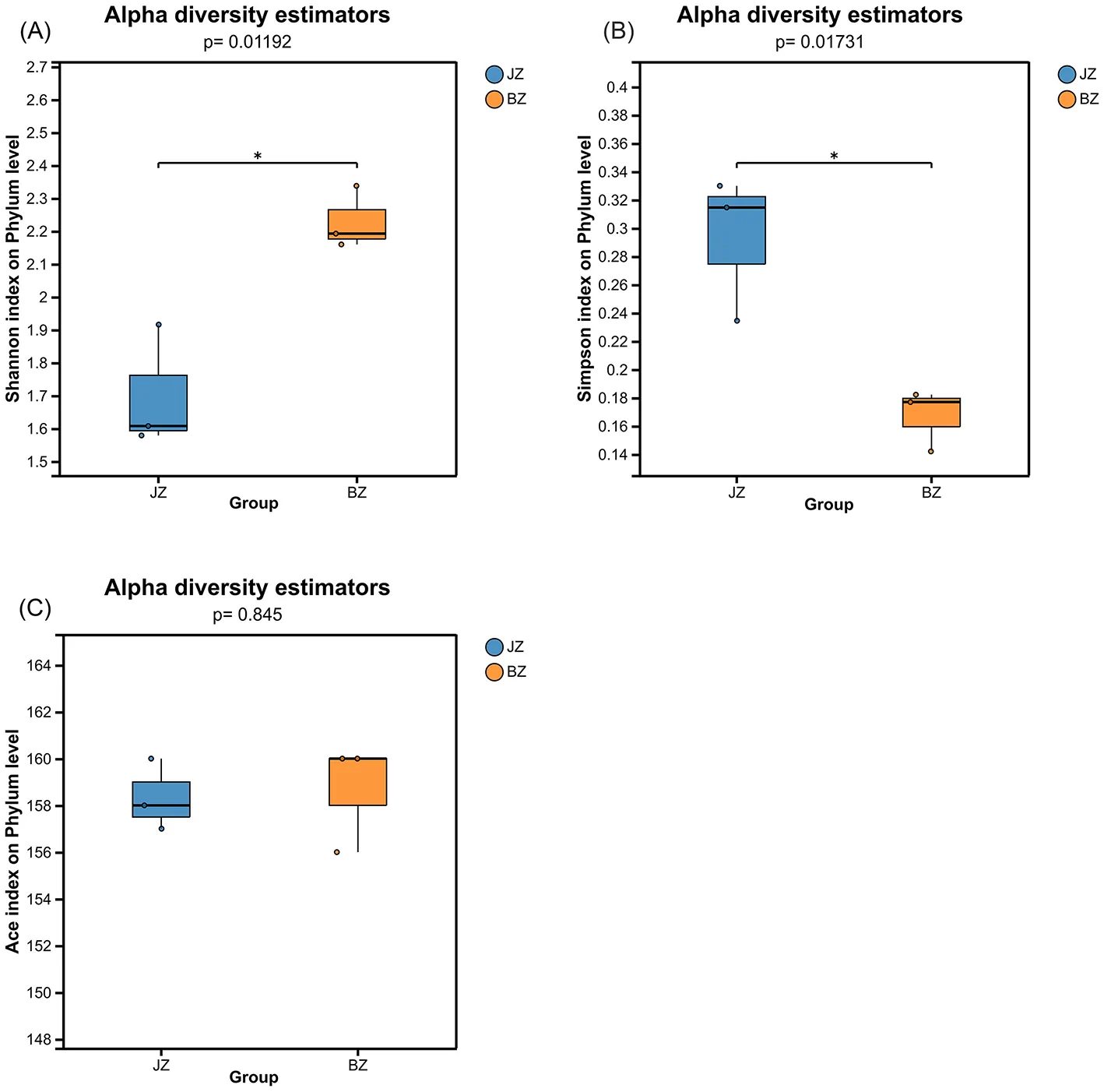
Rhizosphere bacterial alpha-diversity of healthy and diseased *Atractylodes lancea* (*n* = 3 per group). **(A)** Shannon index; **(B)** Simpson's index; **(C)** ACE index. A two-tailed student's *t*-test with FDR multiple testing correction was used for intergroup comparison. Statistical significance was defined according to a threshold of corrected *P* < 0.05. JZ, healthy group; BZ, diseased group.

The coverage index of all the samples reached 1.00, which indicated that the sequencing depth was sufficient to accurately reflect the actual diversity of bacteria in the rhizosphere soil. At the phylum level, alpha diversity analysis revealed obvious differences between the healthy group (JZ) and diseased group (BZ; Figure 1). As shown in Figure 1A, the Shannon index of the diseased group was significantly higher than that of the healthy group (*P* = 0.01192). In contrast, Simpson's index of the diseased group decreased significantly compared with the healthy group (*P* = 0.01731, Figure 1B). The ACE index, which reflects community species richness, exhibited no remarkable statistical difference between the two groups (*P* = 0.845, Figure 1C). These findings suggest that the rhizosphere bacterial community was more diverse in the diseased *A. lancea* plants (Figure 1).

#### 3.2.2 Beta-diversity

**Figure 2:**
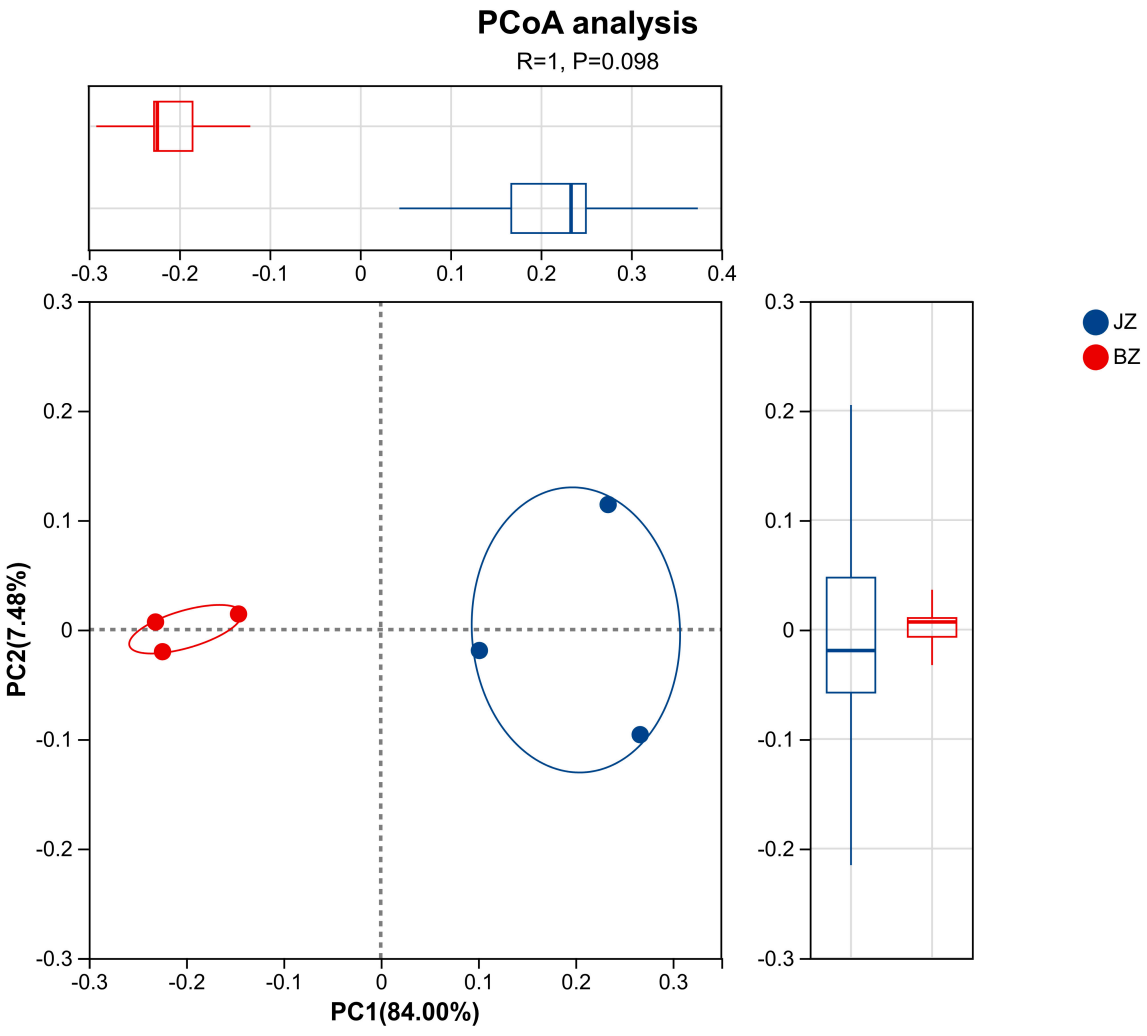
Principal coordinate analysis of rhizosphere bacteria in healthy and diseased *Atractylodes lancea* (*n* = 3 per group). Bray–Curtis distance was adopted for principal coordinate analysis, and ANOSIM test was used to evaluate intergroup dissimilarity, with statistical significance defined according to a threshold of *P* < 0.05. JZ, healthy group; BZ, diseased group.

A principal coordinate analysis (PCoA) revealed that there was a clear separation of the rhizosphere bacterial communities between the healthy and diseased groups along the PC1 axis. There was no overlap of confidence ellipses between the two sample groups (Figure 2). The PC1 and PC2 axes explained 84.00% and 7.48% of the total community variation, respectively. Thus, they represent the primary drivers of bacterial community differentiation. ANOSIM analysis yielded *R* = 1 and *P* = 0.098, indicating obvious divergence in the composition of rhizosphere bacterial communities between diseased and healthy *A*. *lancea*.

### 3.3 Composition and differential analysis of the rhizosphere bacterial communities

#### 3.3.1 Community composition and differences at the phylum level

**Figure 3:**
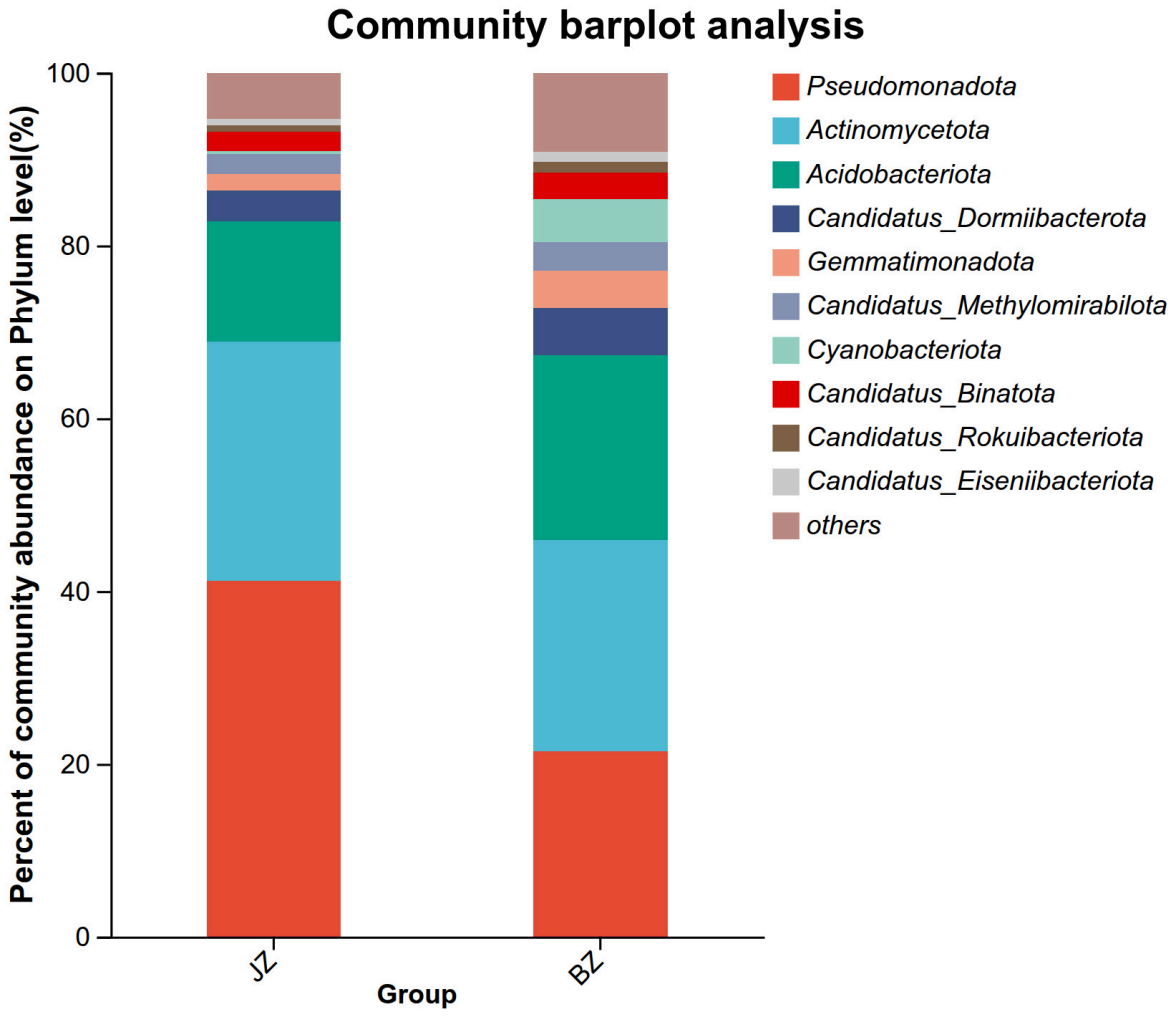
Phylum-level composition of rhizosphere bacterial communities in healthy and diseased *Atractylodes lancea* (*n* = 3 per group). Relative abundance was calculated using the RPKM method. The top 10 phyla ranked by abundance are presented, and the remaining taxa with low abundance are combined into Others. JZ, healthy group; BZ, diseased group.

Six bacterial phyla with relative abundances >2% were identified in the rhizosphere soil of healthy *A. lancea* plants, including *Pseudomonadota* (41.17%), *Actinomycetota* (27.67%), *Acidobacteriota* (13.97%), *Candidatus* Dormibacterota (3.52%), *Candidatus* Methylomirabilota (2.39%), and *Candidatus* Binatota (2.26%). Eight bacterial phyla in the diseased group had a relative abundance > 2%, namely *Actinomycetota* (24.45%), *Acidobacteriota* (21.43%), *Pseudomonadota* (21.41%), *Candidatus* Dormibacterota (5.44%), *Cyanobacteriota* (5.01%), *Gemmatimonadota* (4.34%), *Candidatus* Methylomirabilota (3.27%), and *Candidatus* Binatota (3.09%). Among them, *Pseudomonadota, Actinomycetota*, and *Acidobacteriota* were the dominant phyla with relative abundances each exceeding 10% individually. Collectively, they accounted for more than 50% of the total abundance of bacteria (Figure 3).

**Figure 4:**
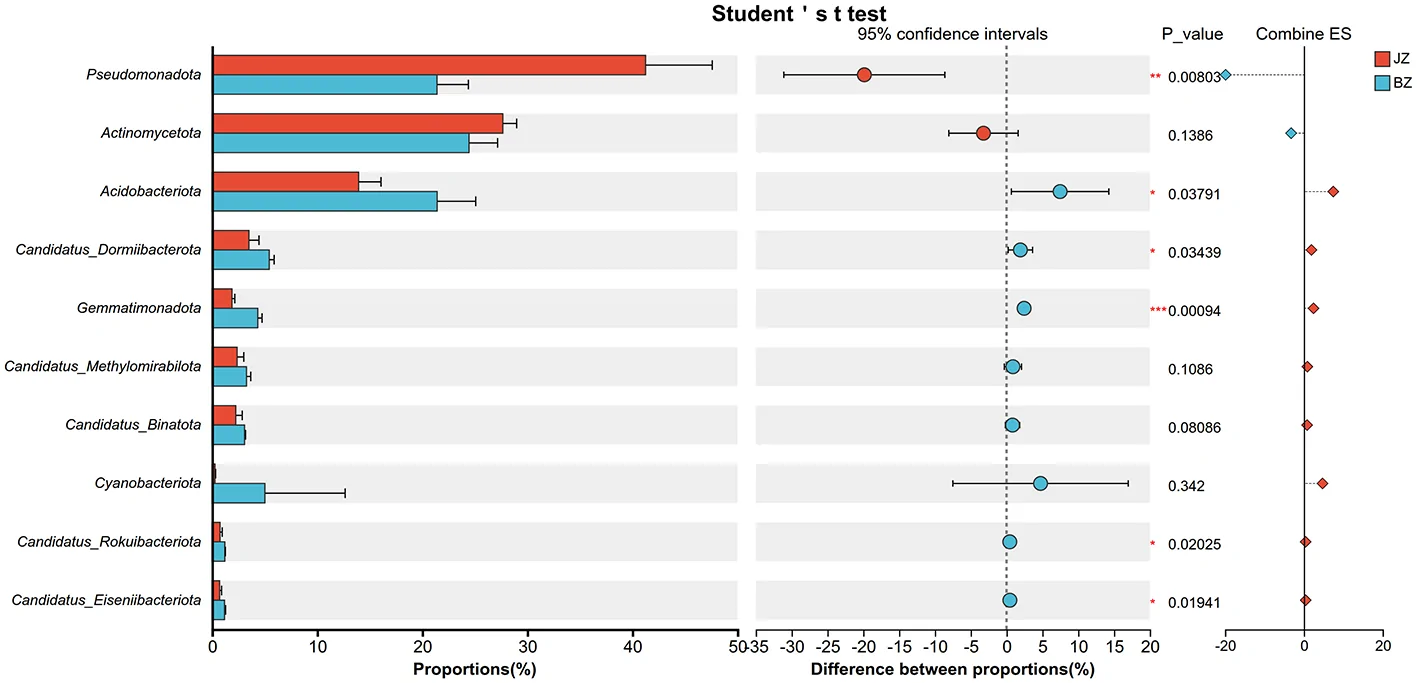
Differences in phylum-level bacterial relative abundance between healthy and diseased *Atractylodes lancea* (*n* = 3 per group). Relative abundance was calculated using the RPKM method. Intergroup differences were assessed by two-tailed Student's *t*-test with FDR correction, and corrected *P* < 0.05 was defined as statistically significant. JZ, healthy group; BZ, diseased group.

The differential analysis indicated that *Pseudomonadota* was significantly more abundant in the healthy group (*P* = 0.00803). In contrast, the diseased group exhibited significantly higher relative abundances of *Acidobacteriota* (*P* = 0.03791), *Candidatus* Dormibacterota (*P* = 0.03439), *Gemmatimonadota* (*P* = 0.00094), *Candidatus* Rokuibacteriota (*P* = 0.02025), and *Candidatus* Eiseniibacteriota (*P* = 0.01941; Figure 4). Several other phyla, including *Actinomycetota*, displayed no statistically meaningful difference in relative abundance between the two treatments (*P* > 0.05). These significant differences in the relative abundance of major bacterial phyla demonstrate clear compositional divergence in rhizosphere bacterial communities between healthy and diseased *A*. *lancea*.

#### 3.3.2 Community composition and differences at the genus level

**Figure 5:**
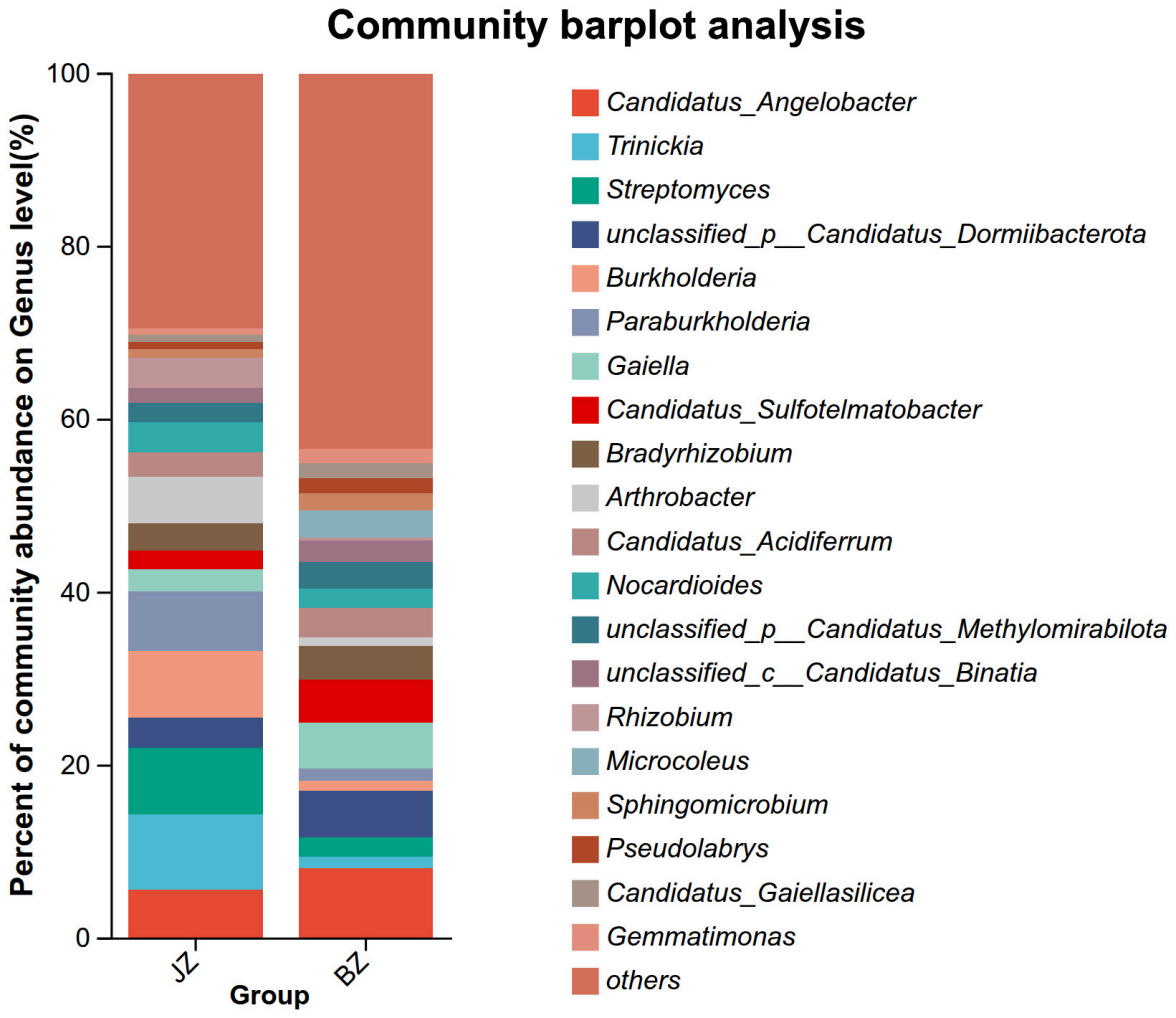
Genus-level composition of rhizosphere bacterial communities in healthy and diseased *Atractylodes lancea* (*n* = 3 per group). Relative abundance was calculated using the RPKM method. The top 20 genera ranked by abundance are presented, and the remaining taxa with low abundance are combined into Others. JZ, healthy group; BZ, diseased group.

A total of 14 bacterial genera with relative abundance >2% were detected in the samples of soil from the healthy rhizosphere soil, including *Trinickia* (8.76%), *Streptomyces* (7.71%), *Burkholderia* (7.71%), *Paraburkholderia* (6.88%), *Candidatus* Angelobacter (5.51%), *Arthrobacter* (5.38%), unclassified_p_*Candidatus* Dormibacterota (3.48%), *Rhizobium* (3.47%), *Nocardioides* (3.46%), *Bradyrhizobium* (3.19%), *Candidatus* Acidiferrum (2.79%), *Gaiella* (2.58%), unclassified_p_*Candidatus* Methylomirabilota (2.25%), and *Candidatus* Sulfotelmatobacter (2.12%; Figure 5).

A total of 12 bacterial genera that had a relative abundance >2% were identified in the diseased rhizosphere soil samples, including *Candidatus* Angelobacter (8.01%), unclassified_p_*Candidatus* Dormibacterota (5.37%), *Gaiella* (5.32%), *Candidatus* Sulfotelmatobacter (4.96%), *Bradyrhizobium* (3.88%), *Candidatus* Acidiferrum (3.39%), *Microcoleus* (3.15%), unclassified_p_*Candidatus* Methylomirabilota (3.08%), unclassified_c_*Candidatus* Binatia (2.42%), *Nocardioides* (2.27%), *Streptomyces* (2.23%), and *Sphingomicrobium* (2.02%; Figure 5).

**Figure 6:**
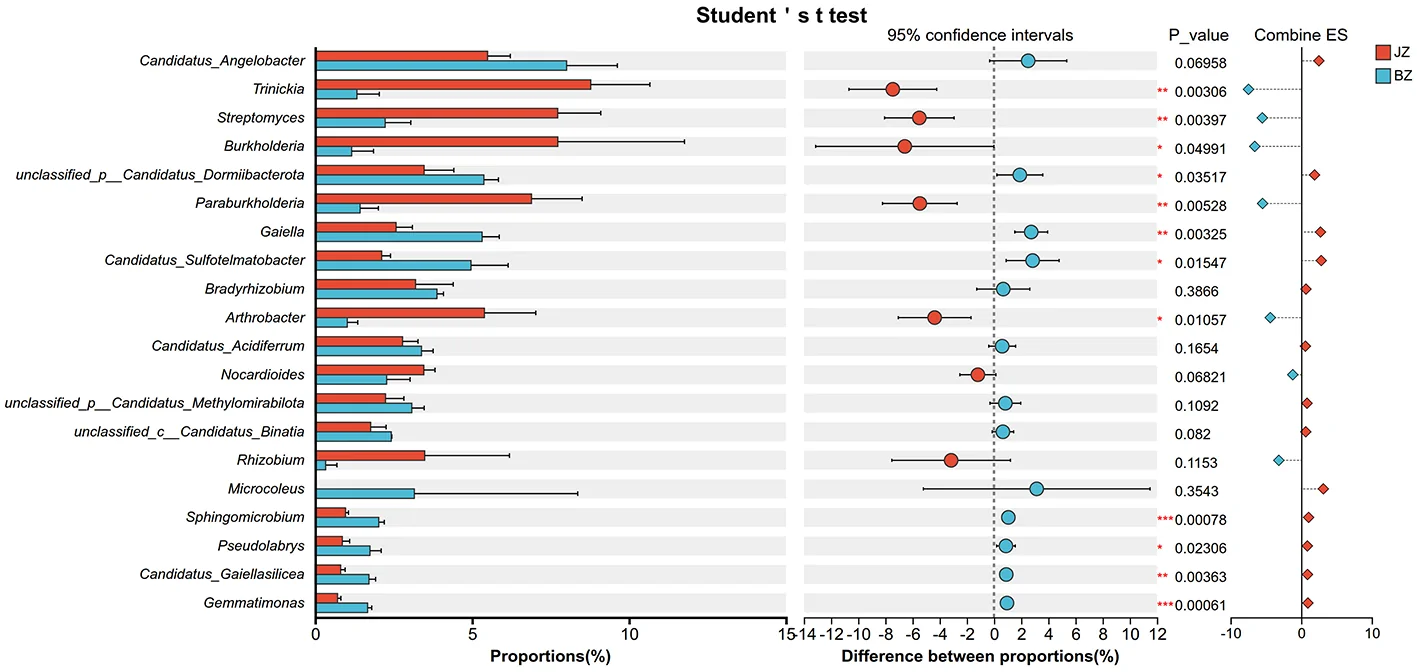
Differences in genus-level bacterial relative abundance between healthy and diseased *Atractylodes lancea* (*n* = 3 per group). Relative abundance was calculated using the RPKM method. Intergroup differences were assessed by two-tailed Student's *t*-test with FDR correction, and corrected *P* < 0.05 was defined as statistically significant. JZ, healthy group; BZ, diseased group.

The differential analysis demonstrated that the relative abundances of *Trinickia* (*P* = 0.00306), *Streptomyces* (*P* = 0.00397), *Burkholderia* (*P* = 0.04991), *Paraburkholderia* (*P* = 0.00528), and *Arthrobacter* (*P* = 0.01057) were each significantly higher in the healthy group. Conversely, the diseased group had significantly higher abundances of unclassified_p_*Candidatus* Dormibacterota (*P* = 0.03517), *Gaiella* (*P* = 0.00325), *Candidatus* Sulfotelmatobacter (*P* = 0.01547), *Sphingomicrobium* (*P* = 0.00078), *Pseudolabrys* (*P* = 0.02306), *Candidatus* Gaiellasilicea (*P* = 0.00363), and *Gemmatimonas* (*P* = 0.00061; Figure 6). Several other genera, including *Bradyrhizobium*, displayed no statistically meaningful difference in relative abundance between the two treatments (*P* > 0.05). These differences in dominant genera at the genus level further demonstrate the obvious compositional divergence of rhizosphere bacterial communities between healthy and diseased individuals.

#### 3.3.3 LEfSe analysis of the differential bacterial taxa

**Figure 7:**
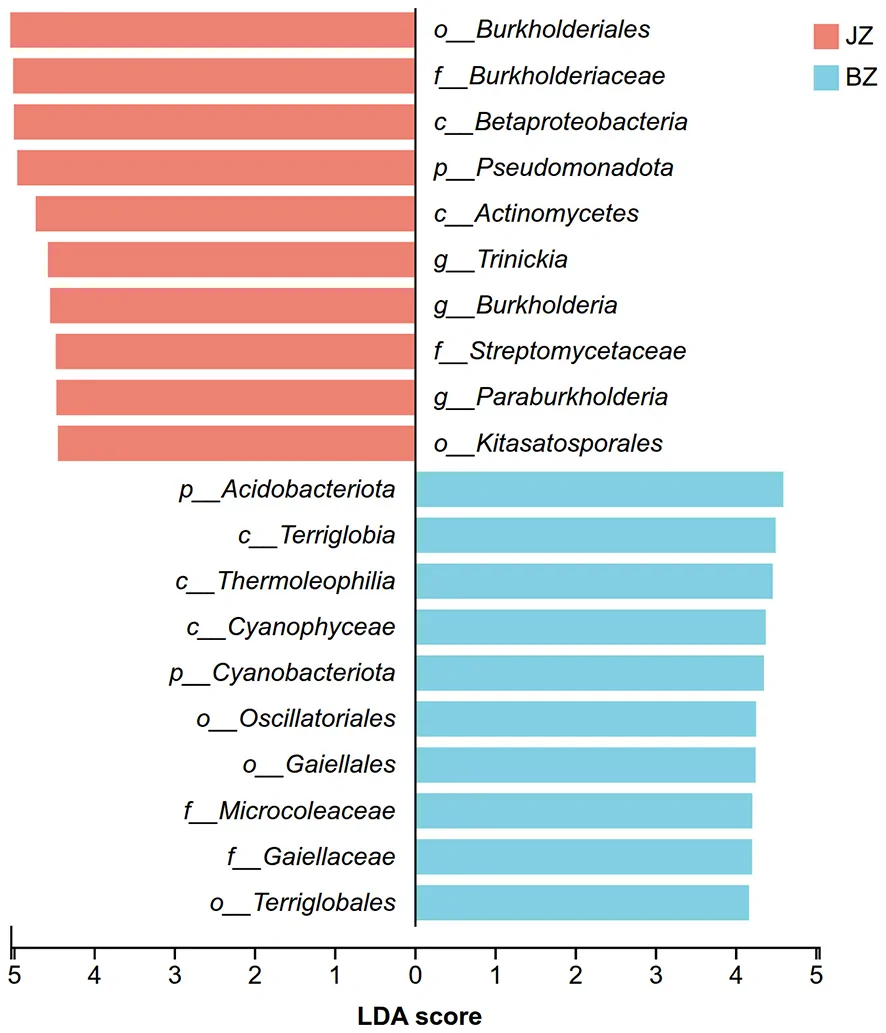
LDA of differential bacterial taxa in healthy and diseased *Atractylodes lancea* (*n* = 3 per group). Relative abundance was calculated using the RPKM method. Taxonomic levels ranging from phylum to genus were screened *via* Wilcoxon rank-sum test, and features with LDA score > 4.5 were regarded as significantly discriminative biomarkers. JZ, healthy group; BZ, diseased group.

The LEfSe analysis (LDA score > 4.0) was performed to screen the biomarker taxa between groups (Figure 7, Table S1). At the phylum level, *Pseudomonadota* was the dominant biomarker of the healthy group, while *Acidobacteriota* and *Cyanobacteriota* were the signature phyla in the diseased group. At the genus level, the core differential genera in the healthy group included *Trinickia, Burkholderia*, and *Paraburkholderia*, which are all members of the family Burkholderiaceae within the phylum *Pseudomonadota*. These characteristic biomarker taxa further typify the taxonomic distinctions of rhizosphere bacterial communities between healthy and root-diseased *A. lancea*.

### 3.4 Functional annotation of the rhizosphere bacterial communities

#### 3.4.1 COG functional annotation

**Figure 8:**
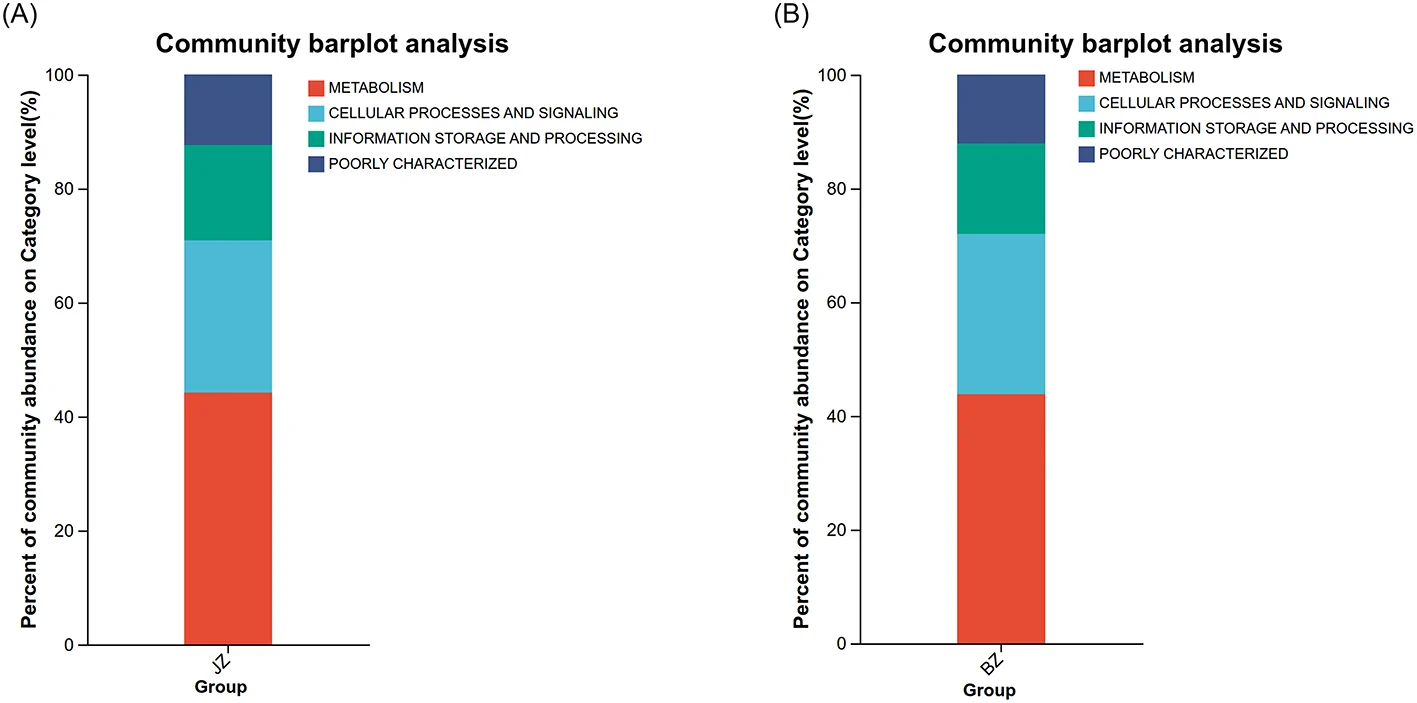
Functional category abundance in healthy and diseased *Atractylodes lancea* (*n* = 3 per group). Relative abundance was quantified using the RPKM method. The top 50 functional categories ranked by abundance are displayed, and the remaining low-abundance entries are merged into Others. JZ, healthy group; BZ, diseased group.

The identified genes were functionally annotated using the COG database. The functional profiles of both the healthy and diseased groups were primarily composed of the following three categories: metabolism, cellular processes and signaling, and information storage and processing (Figure 8). No obvious divergence in the overall proportion distribution of these four primary COG functional categories was observed between healthy and diseased rhizosphere bacterial communities.

#### 3.4.2 Enrichment of the KEGG metabolic pathway

**Figure 9:**
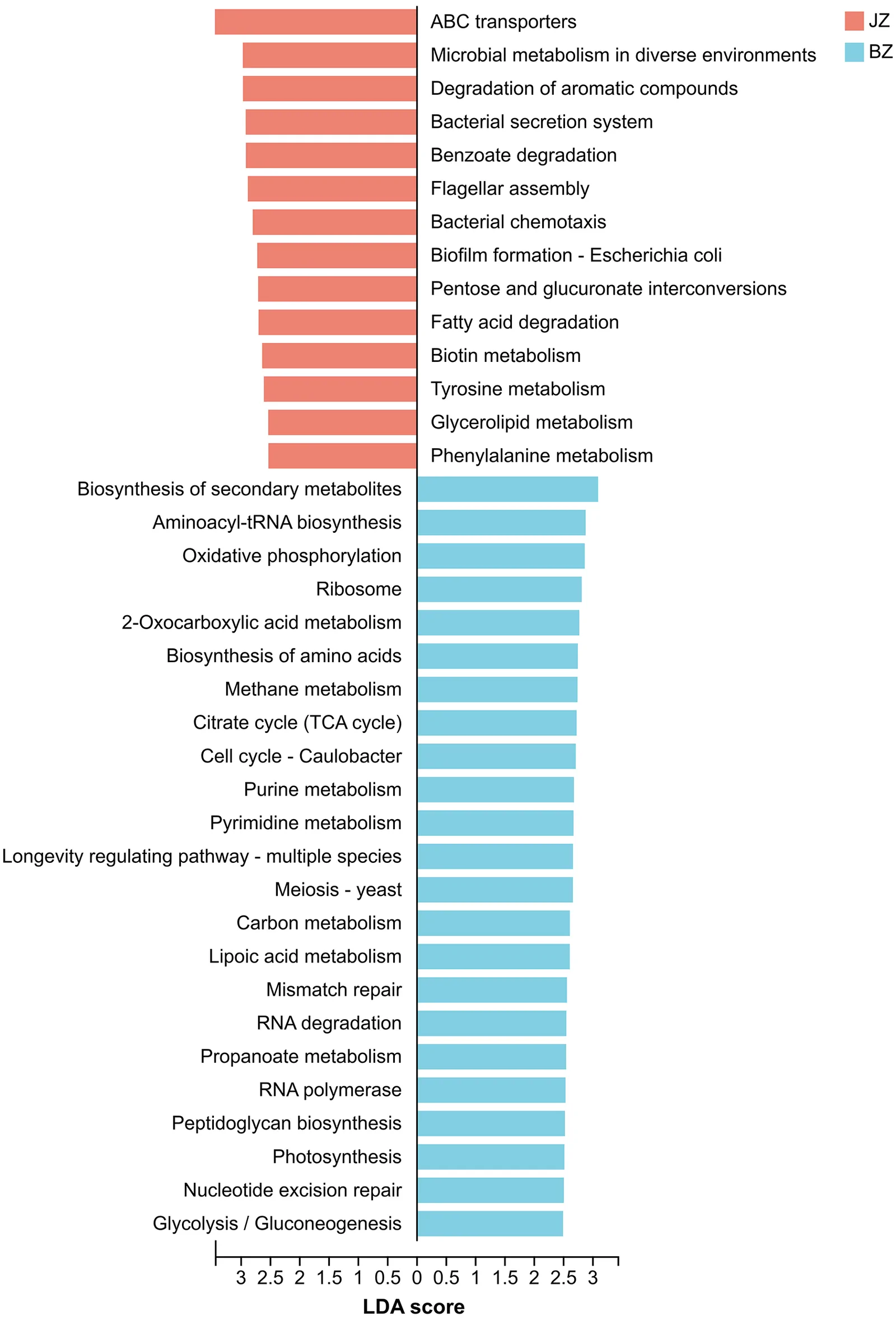
LEfSe analysis of KEGG metabolic pathways in rhizosphere bacterial communities between healthy and diseased *Atractylodes lancea* (*n* = 3 per group). Relative abundance was calculated using the RPKM method. Two-group comparison was conducted *via* Wilcoxon rank-sum test, and pathways with LDA score > 2.5 were screened as significantly differential functional biomarkers. JZ, healthy group; BZ, diseased group.

The KEGG enrichment analysis predicted a total of 37 differential metabolic pathways, including 14 differential pathways enriched in the healthy group and 23 in the diseased group (Figure 9, Table S2). The pathway for the ABC transporters was the core differential metabolic pathway enriched in the healthy communities of rhizosphere bacteria, whereas the biosynthesis of secondary metabolites pathway dominated in the diseased group. Additionally, pathways related to microbial metabolism in diverse environments, degradation of aromatic compounds, and bacterial secretion systems were relatively enriched in the healthy group. In contrast, aminoacyl-tRNA biosynthesis, oxidative phosphorylation, and the ribosome pathways were significantly enriched in the rhizosphere bacteria from the diseased tissue. These divergent enriched KEGG pathways demonstrate the distinct metabolic tendencies of rhizosphere bacterial communities between healthy and diseased *A. lancea*.

#### 3.4.3 CAZy functional annotation

The carbohydrate-active enzymes were subjected to functional annotation prediction using the CAZy database. The relative abundance of carbohydrate-active enzyme families was highest for glycoside hydrolases (GHs, 35.86%), followed by glycosyltransferases (GTs, 34.80%), carbohydrate esterases (CEs, 14.57%), auxiliary oxidoreductases (AAs, 10.13%), polysaccharide lyases (PLs, 2.54%), carbohydrate-binding modules (CBMs, 2.10%), in descending order. A more detailed comparison showed that the overall composition of the carbohydrate-active enzymes was similar between the bacterial communities from the healthy and diseased rhizospheres. Only the abundance of auxiliary oxidoreductases (AAs) differed significantly, and there was a higher relative abundance in the diseased group (Table 2). Such an overall consistent CAZy composition, with only significant elevation of AAs in diseased rhizosphere, indicates minor functional differentiation in carbohydrate metabolism between the two rhizosphere bacterial communities.

**Table 2:** Relative abundance of carbohydrate-active enzymes from rhizosphere bacterial communities of healthy and diseased *Atractylodes lancea*.

Treatment group	Glycoside hydrolases (GHs)%	Glycosyltransferases (GTs)%	Polysaccharide lyases (PLs)%	Carbohydrate-binding modules (CBMs)%	Carbohydrate esterases (CEs) %	Auxiliary activities (AAs)%
JZ	34.32 ± 1.51a	37.07 ± 2.47a	2.83 ± 0.28a	2.01 ± 0.14a	14.29 ± 0.33a	9.47 ± 0.39b
BZ	36.78 ± 1.79a	33.40 ± 1.84a	2.38 ± 0.20a	2.16 ± 0.07a	14.76 ± 0.16a	10.52 ± 0.44a

Data are expressed as mean ± standard deviation values; different lowercase letters within the same column indicate significant differences at P < 0.05. JZ, healthy group; BZ, diseased group.

### 3.5 Redundancy analysis

**Figure 10:**
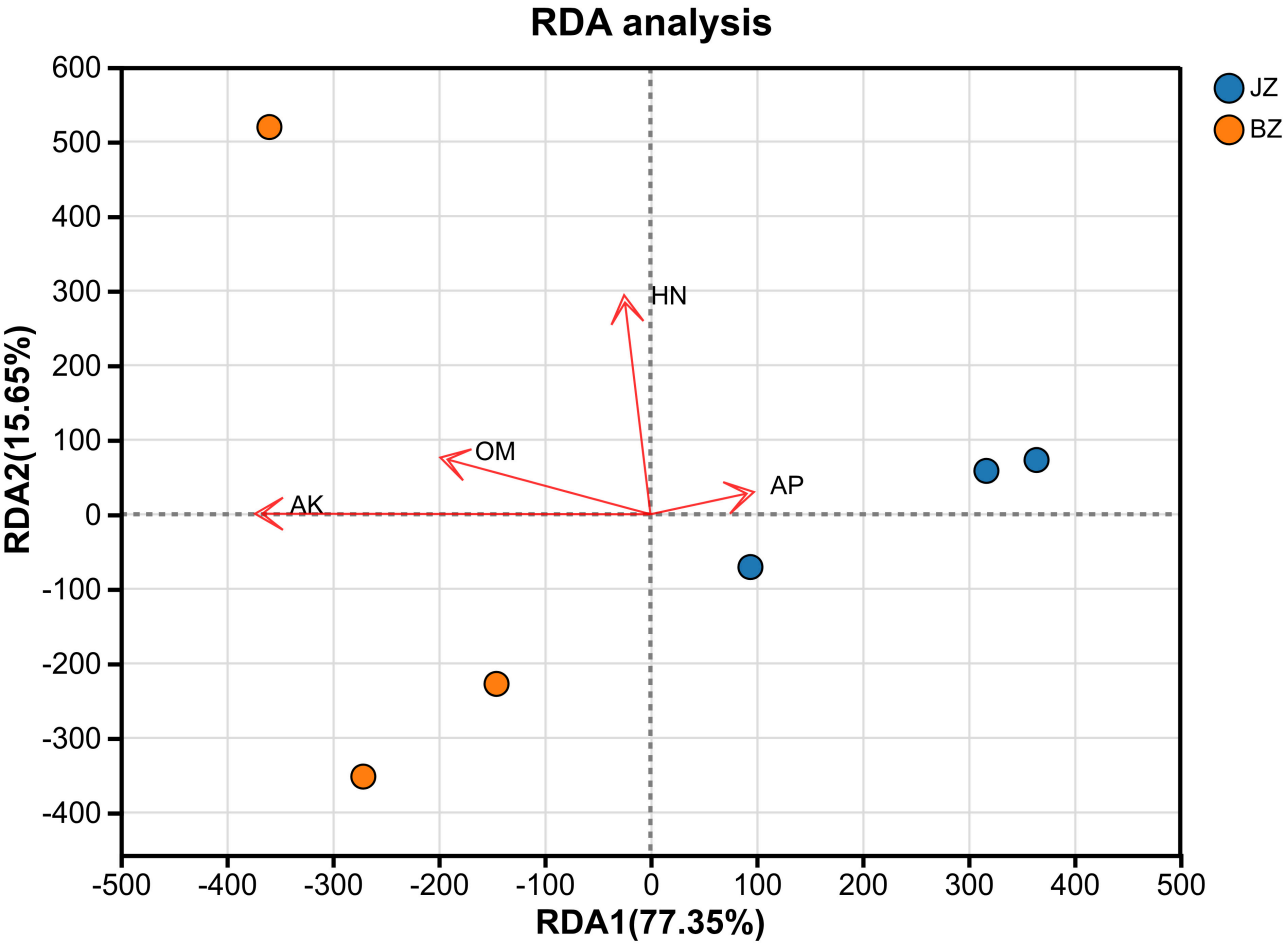
Redundancy analysis of rhizosphere bacteria and soil environmental factors in *Atractylodes lancea* (*n* = 3 per group). OM, organic matter; HN, hydrolyzable nitrogen; AP, available phosphorus; AK, available potassium; JZ, healthy group; BZ, diseased group.

The RDA showed that the first two axes explained 93.00% of the total variation in bacterial community composition (Figure 10). RDA1 accounted for 77.35% of the variation and primarily drove the differentiation in community between the two groups. The diseased samples were distributed on the negative side of the RDA1 axis, while the healthy samples were clustered on the positive side. The Envfit analysis revealed that the AK was significantly and negatively correlated with RDA1 (*r*^2^ = 0.94007, *P* = 0.00556; Table 3). This indicated that AK was the core environmental factor that shaped the rhizosphere bacterial community structure.

**Table 3:** Envfit analysis for RDA environmental variables.

Environmental factor	RDA1	RDA2	*r* ^2^	*P* values
OM	−0.943	0.332	0.285	0.553
HN	−0.040	0.999	0.570	0.239
AP	0.944	0.329	0.063	0.876
AK	−0.999	−0.042	0.940	0.006

OM, organic matter; HN, hydrolyzable nitrogen; AP, available phosphorus; AK, available potassium.

### 3.6 Correlation analysis

**Figure 11:**
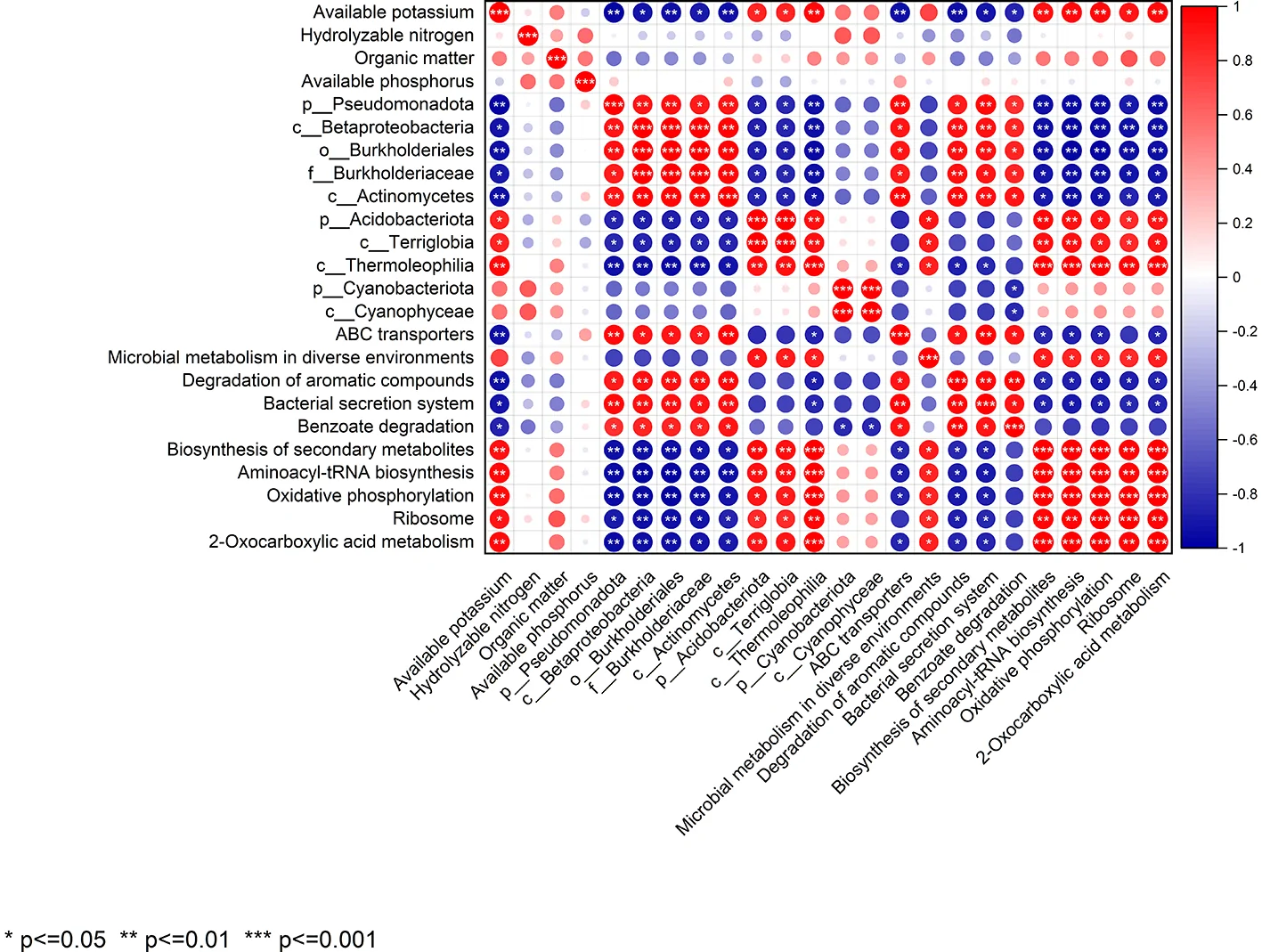
Pearson correlation coefficients between taxa, functions, and soil factors of *Atractylodes lancea* (*n* = 3 per group). *, *P* ≤ 0.05; **, *P* ≤ 0.01; ***, *P* ≤ 0.001.

A Pearson correlation analysis was performed to explore the internal relationships among the soil physicochemical properties, dominant differential bacterial taxa, and microbial metabolic functions. The results showed that the AK in soil was significantly positively correlated with *Acidobacteriota* and c_Thermoleophilia abundances, but significantly negatively correlated with abundances of the taxa in the *Pseudomonadota* (from phylum to family level) and c_Actinomycetes. In terms of metabolic functions, AK was significantly positively correlated with five pathways, including the biosynthesis of secondary metabolites, aminoacyl-tRNA biosynthesis, oxidative phosphorylation, ribosome, and 2-oxocarboxylic acid metabolism. Moreover, AK was significantly negatively correlated with the following four pathways, including ABC transporters, degradation of aromatic compounds, bacterial secretion system, and benzoate degradation (Figure 11). Collectively, these correlation results further illustrate that soil AK acts as a key environmental factor linking the variation of rhizosphere bacterial community composition and metabolic functional profiles during root rot development.

## 4 Discussion

### 4.1 Changes in soil physicochemical properties

In this study, the contents of TN, TK, and AK were significantly higher in the rhizosphere soil of diseased *A. lancea* plants than in that of the healthy plants. These findings are consistent with previous studies on root rot diseases in other medicinal and cash crops. Li et al. (2023) reported higher contents of organic matter and N in the rhizosphere of tobacco (*Nicotiana tabacum*) infected with an microbe that causes root rot. He et al. (2026) also observed an increase in the levels of TN in the rhizosphere soil from diseased *Hevea brasiliensis*.

Soil is highly heterogeneous in its physical and chemical properties. Thus, it provides diverse ecological niches for microorganisms and constitutes one of the most microbially abundant ecosystems on Earth. It is widely regarded as a seed bank for microbial communities in the rhizosphere (Ling et al., 2022). Complex physicochemical characteristics of the soil directly shape the composition of soil microbial communities and drive the initial assembly of microorganisms, thereby determining the final structure of rhizosphere microbiomes (Jeong et al., 2024). Plant roots can actively regulate rhizosphere microenvironments by secreting specific metabolites in response to nutrient limitations. For example, legumes secrete flavonoids when the soil is deficient in N to recruit rhizobia and activate the genes related to nodulation; this response ultimately establishes symbiotic relationships with bacteria on the roots (Peters et al., 1986). Similarly, *P* starvation prompts *Arabidopsis* to respond in ways that facilitate the acquisition of P by inducing the expression of genes associated with the biosynthesis of organic acids and glucosinolates (Castrillo et al., 2017).

Limitations of this study should be noted; for example, several pivotal soil physicochemical variables, including pH, electrical conductivity, soil moisture, and soil organic carbon, were not quantified, which restricts further interpretation of environmental filtering effects on rhizosphere bacterial communities. Follow-up research will integrate these environmental indicators to clarify their regulatory roles in microbiota variation.

### 4.2 Shifts in bacterial alpha and beta diversity

In the present study, bacterial diversity in rhizosphere soil was significantly higher in diseased *A. lancea* than in healthy individuals. The PCoA results further confirmed the clear structural separation between the healthy and diseased microbial communities, which is consistent with earlier findings on the microbial differentiation induced by soilborne diseases (Chen et al., 2024; Wei et al., 2024).

Pathogen infection status has been reported to be linked to marked differences in rhizosphere microbial community structure between diseased and healthy plants (Wang et al., 2024). Previous studies have verified that dynamic shifts in rhizosphere microbial composition enable early diagnosis of tomato bacterial wilt (Gu et al., 2022). Similarly, the outbreak of diseases in *Panax notoginseng* is closely associated with shifts in rhizosphere soil microbial diversity (Liu Y. et al., 2024). Nevertheless, no significant difference in rhizosphere bacterial alpha diversity was detected between *Cotinus coggygria* plants infected with *Verticillium* wilt and healthy plants (Zhao et al., 2024). Deng et al. (2022) reported that rhizosphere microbial diversity of *Pinus koraiensis* increased markedly with aggravated pine wilt disease symptoms. Such inconsistent findings among different studies are most likely attributed to disparities in plant species, disease severity, sampling-period temperature, and soil characteristics (Sun et al., 2025; Xiang et al., 2023).

### 4.3 Alterations of bacterial community composition at phylum and genus levels

This study showed that *Pseudomonadota, Actinomycetota*, and *Acidobacteriota* dominated the rhizosphere bacterial communities of both healthy and diseased *A. lancea* plants, which is consistent with the findings of previous studies on the microorganisms in its rhizosphere. Hu et al. (2021) reported that *Actinomycetota, Acidobacteriota, Pseudomonadota*, and *Gemmatimonadota* constituted the core rhizosphere bacterial flora of Chinese cabbage. These findings support the pervasiveness of these phyla in the rhizosphere of crop plants. The microbial community structure of the rhizosphere is essential to maintain plant physiological metabolism, enhance stress tolerance, and resist pathogen invasions (Philippot et al., 2013; Yue et al., 2025). As such, changes in the composition and abundance of microorganisms are tightly linked to the occurrence of disease (Kong et al., 2022).

In this study, the diseased rhizosphere soil exhibited a significant enrichment of *Acidobacteriota* and *Gemmatimonadota* and depletion of *Pseudomonadota*, which is consistent with previous observations on root rot in tobacco (Liu H. et al., 2024). As a common soil bacterial phylum that is highly adaptable to the environment and flexible metabolically, *Gemmatimonadota* actively participates in soil carbon, *N*, and P cycling and is highly sensitive to fluctuations in nutrients; thus, it is a reliable bioindicator of soil environmental changes (Mujakić et al., 2022). Previous studies have demonstrated that the abundance of *Gemmatimonas* is positively correlated with the concentrations of nutrients in the soil, and their enrichment facilitates its proliferation (Liu et al., 2022a). Combined with the findings of the present study, it can reasonably be inferred that the accumulation of nutrients in the rhizosphere soil induced by root rot promotes the colonization of *Gemmatimonadota* species that prefer nutrients, which further participates in microecological succession under disease stress.

In addition, this study found that Cyanobacteria were significantly enriched in the diseased rhizosphere soil. As typical pioneer microorganisms, *Cyanobacteria* participate in the fixation of *N* and activation of nutrients in the soil, and they commonly proliferate in degraded and imbalanced soil microenvironments. Soil physicochemical property changes linked to root rot were associated with excessive proliferation of soil *Cyanobacteria*. The excessive proliferation of cyanobacteria may occupy the ecological niche of beneficial microorganisms and, thus, further disrupt the community stability and aggravate microecological imbalances in the rhizosphere (Fang et al., 2025).

The LEfSe analysis revealed distinct biomarker taxa between the healthy and diseased groups. At the genus level, all the key differential biomarkers in the healthy rhizosphere soil belonged to the family Burkholderiaceae. *Burkholderia* are ubiquitous beneficial soil bacteria with multiple ecological functions, including the degradation of pollutants, promotion of plant growth, and bioremediation of soil (Carrión et al., 2018). Members of this family can effectively antagonize soilborne pathogens, alleviate disease damage, and sustain microecological homeostasis in the rhizosphere (Luo et al., 2021). Their antagonistic mechanisms include the secretion of bioactive compounds that induce the accumulation of reactive oxygen species and subsequent oxidative damage in pathogens, thereby inhibiting their colonization and infection (Ayaz et al., 2021). Furthermore, *Burkholderia* can suppress the proliferation of pathogens by reshaping the rhizosphere community structure and balancing microbial interactions (Cao et al., 2022; Liu et al., 2022b). They also enhance systemic resistance in plants by activating the defense pathway mediated by salicylic acid, which improves the tolerance of plants to biotic stress (Tian et al., 2022).

Accordingly, the enrichment of Burkholderiaceae in healthy rhizosphere soil appears to contribute to stable microecological conditions and effective disease suppression. In contrast, reductions in the abundance of these beneficial taxa in diseased rhizospheres weaken rhizosphere pathogen resistance and promote root rot progression (Kim et al., 2023). Notably, although Burkholderiaceae species are generally considered plant-beneficial rhizobacteria, this family also contains opportunistic pathogenic species, such as the *Burkholderia cepacia* complex and *B*. *pseudomallei*. Therefore, their ecological roles are context-dependent, and further species-level identification is required to precisely clarify their functions in the *A. lancea* rhizosphere microecosystem.

### 4.4 Functional characteristics of rhizosphere microbiota based on COG, KEGG, and CAZy annotations

Functional annotation based on COG revealed that functional potential of microbial genes in both healthy and diseased rhizosphere soils were predominantly categorized into metabolism, cellular processes and signaling, and information storage and processing. This functional distribution is consistent with the general characteristics of plant rhizosphere microbiomes and reflects the basic functional stability of the rhizosphere ecosystem of *A. lancea* (Peng et al., 2024).

KEGG enrichment analysis in this study comprehensively revealed distinct metabolic differentiation in functional potential between healthy and diseased rhizosphere bacterial communities. Specifically, the rhizosphere bacteria of healthy *A. lancea* plants were significantly enriched in annotations for ABC transporter pathways, whereas the microbiota in diseased rhizosphere soil exhibited marked enrichment of amino acid biosynthesis pathways. ABC transporters are widely distributed across prokaryotic and eukaryotic organisms, and they mediate the ATP-dependent uptake of nutrients, transport of metabolites, and metabolism of secondary substances (Bilsing et al., 2023). They also regulate the translocation of intracellular material and enhance the adaptability of plants and their ability to resist pathogens under complex soil conditions (Banasiak and Jasiński, 2022). Amino acid biosynthesis pathways are closely involved in microbial biofilm formation, protein synthesis, and cell proliferation. Rhizosphere microenvironmental differences between diseased and healthy plants may be associated with altered bacterial amino acid metabolism, potentially supporting microbial growth, community reproduction, and environmental adaptation under stress conditions (Xu et al., 2022).

CAZy annotation results further indicated that the genes encoding auxiliary oxidoreductases (AAs) were more abundant in diseased rhizosphere soil. Auxiliary oxidoreductases participate in the oxidative transformation of recalcitrant organic matter and provide active oxygen for the degradation of lignocellulose, which plays vital roles in the microbial decomposition of complex soil organic compounds (Levasseur et al., 2013). These predictive results imply that enhanced organic matter degradation metabolism of rhizosphere microorganisms is associated with microenvironmental shifts linked to root rot pathogen infection.

### 4.5 AK acts as a key environmental driver reshaping rhizosphere bacterial communities and functions

In the present study, significant potassium accumulation was detected in the rhizosphere soil of diseased *A*. *lancea*. Potassium plays a critical role in enhancing disease resistance and increasing the tolerance of plants to stress, and appropriate fertilization with potassium improves the ability of plants to defend against soilborne pathogens, such as *Fusarium* (Cai et al., 2020). An increase in the AK in diseased rhizosphere soil has been frequently documented in previous studies on root rots and wilts. Yang et al. (2023) found that the contents of AK significantly accumulated in the rhizosphere of cucumber (*Cucumis sativus*) under *Fusarium* wilt stress. Fan et al. (2024) similarly reported higher levels of AK in the rhizosphere soil of diseased *Atractylodes macrocephala*. Variation in the physicochemical properties of the soil substantially disturbs the rhizosphere microbial assembly and exacerbates the development of soilborne diseases (Liao et al., 2026; Liu et al., 2026). Generally, soil AK content is positively correlated with disease severity, further indicating that changes in soil potassium conditions are closely associated with the progression of root rot disease.

In this study, the content of AK was significantly correlated with the relative abundance of multiple differential bacterial taxa and thus seems to have substantially reshaped the microbial metabolic profiles. In particular, it is associated with the biosynthesis of secondary metabolites. Diseased rhizosphere microbiota enriched the functions for the biosynthesis of secondary metabolites. This may enhance the adaptability of microorganisms and increase the resistance to stress when plant diseases induce microecological disturbances (Shi et al., 2023). Driven by altered soil nutrient conditions, rhizosphere microorganisms develop complex competitive and synergistic interactions. Microbial communities optimize the allocation of resources and metabolism of energy to improve their ecological adaptability and maintain a stable population under changing rhizosphere environments (Philippot et al., 2024). The availability of nutrients in the soil strongly modulates the structure and function of the bacterial communities in soil (Gao et al., 2022).

Pearson correlation analysis showed that AK was closely associated with the relative abundance of dominant *Pseudomonadota* and *Acidobacteriota*, suggesting that potassium content variation may be associated with community succession. Such shifts in rhizomicrobial assembly associated with altered soil nutrient status are a general feature of plant root zones and can serve as a foundation for soil pollution remediation *via* rhizoremediation (Aryal, 2024).

## 5 Conclusions

This study systematically investigated the relationships among the soil physicochemical properties, rhizosphere bacterial community dynamics, and metabolic functional variation during the occurrence of root rot in *Atractylodes lancea*. Such infections were associated with elevated contents of total nitrogen, total potassium, and AK in soil, higher rhizosphere bacterial diversity, and obvious structural differentiation of microbial communities. The healthy rhizosphere was predominantly enriched with beneficial Burkholderiaceae taxa within *Pseudomonadota*, whereas the diseased rhizosphere soil accumulated opportunistic taxa, including *Cyanobacteriota* and *Gemmatimonadota*. Functionally, predicted functional annotation indicated that healthy rhizosphere microbiota exhibited relative enrichment of ABC transporter pathways, which may be associated with stable nutrient transport and plant disease resistance. In contrast, diseased rhizosphere bacteria showed enrichment in amino acid biosynthesis and auxiliary oxidoreductase-related metabolic pathways, implying potential metabolic adaptation associated with disease-associated rhizosphere microenvironments. AK was found to be strongly associated with shifts in bacterial community composition and functional differentiation. Root rot disease was linked to comprehensive shifts in soil nutrient conditions, microbial community structure, and microbial metabolic functions, which were associated with rhizosphere microecological imbalance. These findings clarify the microecological mechanisms that underlie root rot in *A. lancea* and provide a foundation for the green prevention and sustainable cultivation of medicinal materials.

This study has several limitations, however. The individual rhizosphere samples were pooled into three biological replicates per group, which may partially mask biological variation and reduce statistical robustness. As a field-based investigation, the current study only revealed correlative associations among soil properties, microbial communities, and disease status without confirming definitive causal relationships, as shifts in soil AK and rhizosphere microbiota may either contribute to root rot occurrence or result from plant physiological deterioration after infection. Additionally, the pathogenic strain causing root rot was not isolated and identified in this field sampling, which limits the mechanistic interpretation of microbiome alterations related to root rot. Future work will increase independent biological replicates, include pathogen isolation and identification, and perform controlled experiments and long-term field monitoring to further verify the causal mechanisms underlying rhizosphere microecological variation and disease occurrence.

## Data Availability

The metagenomic data in this study have been submitted to the NCBI database and specifically deposited in the NCBI BioProject platform, with the accession number PRJNA1470840. The accession numbers corresponding to each sample sequence are SRR38847786-SRR38847791.
