# Supplementary material for: Metagenomic sequencing reveals structural and functional differentiation of the rhizosphere bacterial communities associated with available potassium in Atractylodes lancea affected with root rot
Source: Front Microbiol. 2026 Aug 19;17:1923567. doi: 10.3389/fmicb.2026.1923567 (PMC13533974; doi:10.3389/fmicb.2026.1923567)
Supplement: Supplementary file 1 [file Table_1.docx]

Table S1 LDA of differential bacterial taxa in healthy and diseased *Atractylodes lancea*

| Species name | Group | Mean relative abundance | LDA score |
| --- | --- | --- | --- |
| *o__Burkholderiales* | JZ | 5.416 | 5.040 |
| *f__Burkholderiaceae* | JZ | 5.382 | 5.005 |
| *c__Betaproteobacteria* | JZ | 5.432 | 4.995 |
| *p__Pseudomonadota* | JZ | 5.615 | 4.954 |
| *c__Actinomycetes* | JZ | 5.349 | 4.723 |
| *g__Trinickia* | JZ | 4.944 | 4.571 |
| *g__Burkholderia* | JZ | 4.888 | 4.543 |
| *f__Streptomycetaceae* | JZ | 4.895 | 4.474 |
| *g__Paraburkholderia* | JZ | 4.839 | 4.465 |
| *o__Kitasatosporales* | JZ | 4.895 | 4.446 |
| *p__Acidobacteriota* | BZ | 5.331 | 4.601 |
| *c__Terriglobia* | BZ | 5.286 | 4.505 |
| *c__Thermoleophilia* | BZ | 5.030 | 4.469 |
| *c__Cyanophyceae* | BZ | 4.693 | 4.383 |
| *p__Cyanobacteriota* | BZ | 4.701 | 4.359 |
| *o__Oscillatoriales* | BZ | 4.597 | 4.262 |
| *o__Gaiellales* | BZ | 4.847 | 4.256 |
| *f__Microcoleaceae* | BZ | 4.542 | 4.215 |
| *f__Gaiellaceae* | BZ | 4.847 | 4.212 |
| *o__Terriglobales* | BZ | 4.777 | 4.174 |

Note: *n* = 3 per group. JZ, healthy group; BZ, diseased group.
