# Supplementary material for: Metagenomic sequencing reveals structural and functional differentiation of the rhizosphere bacterial communities associated with available potassium in Atractylodes lancea affected with root rot
Source: Front Microbiol. 2026 Aug 19;17:1923567. doi: 10.3389/fmicb.2026.1923567 (PMC13533974; doi:10.3389/fmicb.2026.1923567)
Supplement: Supplementary file 2 [file Table_2.docx]

Table S2 LEfSe analysis of KEGG metabolic pathways in rhizosphere bacterial communities between healthy and diseased *Atractylodes lancea*

| Function name | Group | Meanrelative abundance | LDA score |
| --- | --- | --- | --- |
| ABC transporters | JZ | 4.362 | 3.434 |
| Microbial metabolism in diverse environments | JZ | 4.747 | 2.958 |
| Degradation of aromatic compounds | JZ | 3.763 | 2.955 |
| Bacterial secretion system | JZ | 3.759 | 2.908 |
| Benzoate degradation | JZ | 3.806 | 2.906 |
| Flagellar assembly | JZ | 3.558 | 2.872 |
| Bacterial chemotaxis | JZ | 3.633 | 2.789 |
| Biofilm formation - Escherichia coli | JZ | 3.628 | 2.712 |
| Pentose and glucuronate interconversions | JZ | 3.491 | 2.697 |
| Fatty acid degradation | JZ | 3.772 | 2.690 |
| Biotin metabolism | JZ | 3.571 | 2.628 |
| Tyrosine metabolism | JZ | 3.639 | 2.599 |
| Glycerolipid metabolism | JZ | 3.525 | 2.525 |
| Phenylalanine metabolism | JZ | 3.731 | 2.521 |
| Biosynthesis of secondary metabolites | BZ | 4.876 | 3.103 |
| Aminoacyl-tRNA biosynthesis | BZ | 3.897 | 2.890 |
| Oxidative phosphorylation | BZ | 4.069 | 2.875 |
| Ribosome | BZ | 3.886 | 2.824 |
| 2-Oxocarboxylic acid metabolism | BZ | 3.973 | 2.782 |
| Biosynthesis of amino acids | BZ | 4.361 | 2.758 |
| Methane metabolism | BZ | 3.888 | 2.751 |
| Citrate cycle (TCA cycle) | BZ | 3.838 | 2.736 |
| Cell cycle - Caulobacter | BZ | 3.703 | 2.722 |
| Purine metabolism | BZ | 4.090 | 2.690 |
| Pyrimidine metabolism | BZ | 3.804 | 2.684 |
| Longevity regulating pathway - multiple species | BZ | 3.516 | 2.674 |
| Meiosis - yeast | BZ | 3.371 | 2.673 |
| Carbon metabolism | BZ | 4.412 | 2.620 |
| Lipoic acid metabolism | BZ | 3.620 | 2.620 |
| Mismatch repair | BZ | 3.601 | 2.572 |
| RNA degradation | BZ | 3.623 | 2.560 |
| Propanoate metabolism | BZ | 3.862 | 2.558 |
| RNA polymerase | BZ | 3.337 | 2.547 |
| Peptidoglycan biosynthesis | BZ | 3.665 | 2.537 |
| Photosynthesis | BZ | 3.365 | 2.528 |
| Nucleotide excision repair | BZ | 3.595 | 2.519 |
| Glycolysis / Gluconeogenesis | BZ | 3.961 | 2.505 |

Note: JZ, healthy group; BZ, diseased group.
